# Energy Demands of Early Life Drive a Disease Tolerant Phenotype and Dictate Outcome in Neonatal Bacterial Sepsis

**DOI:** 10.3389/fimmu.2018.01918

**Published:** 2018-08-23

**Authors:** Danny Harbeson, Freddy Francis, Winnie Bao, Nelly A. Amenyogbe, Tobias R. Kollmann

**Affiliations:** ^1^Department of Experimental Medicine, University of British Columbia, Vancouver, BC, Canada; ^2^Department of Pediatrics, Division of Infectious Diseases, University of British Columbia, Vancouver, BC, Canada

**Keywords:** neonate, sepsis, disease tolerance, metabolism, inflammation, infection

## Abstract

Bacterial sepsis is one of the leading causes of death in newborns. In the face of growing antibiotic resistance, it is crucial to understand the pathology behind the disease in order to develop effective interventions. Neonatal susceptibility to sepsis can no longer be attributed to simple immune immaturity in the face of mounting evidence that the neonatal immune system is tightly regulated and well controlled. The neonatal immune response is consistent with a “disease tolerance” defense strategy (minimizing harm from immunopathology) whereas adults tend toward a “disease resistance” strategy (minimizing harm from pathogens). One major advantage of disease tolerance is that is less energetically demanding than disease resistance, consistent with the energetic limitations of early life. Immune effector cells enacting disease resistance responses switch to aerobic glycolysis upon TLR stimulation and require steady glycolytic flux to maintain the inflammatory phenotype. Rapid and intense upregulation of glucose uptake by immune cells necessitates an increased reliance on fatty acid metabolism to (a) fuel vital tissue function and (b) produce immunoregulatory intermediates which help control the magnitude of inflammation. Increasing disease resistance requires more energy: while adults have fat and protein stores to catabolize, neonates must reallocate resources away from critical growth and development. This understanding of sepsis pathology helps to explain many of the differences between neonatal and adult immune responses. Taking into account the central role of metabolism in the host response to infection and the severe metabolic demands of early life, it emerges that the striking clinical susceptibility to bacterial infection of the newborn is at its core a problem of metabolism. The evidence supporting this novel hypothesis, which has profound implications for interventions, is presented in this review.

## Introduction

Improvements in neonatal mortality have been comparatively slower than in other age groups (1). More than 40% of all under-five deaths occur in the neonatal period and this percentage has been rising over the last few decades (1, 2). Part of the difficulties associated with decreasing newborn mortality stems from an assumption that neonatal immunity is “immature” or a “deficient” version of its adult counterpart (3–5). Despite much evidence that debunks this myth (i.e., robust immune responses, heightened sensitivity to sterile inflammatory insults such as LPS, ability to tolerate much higher pathogen load than adults, etc.) (3, 4, 6–9), this dogma persists, likely because it presents an easy explanation for the clinically increased susceptibility (10). Ultimately, the inability of this long-held immaturity paradigm to translate into effective immunomodulatory treatments for neonatal sepsis is in itself evidence of a fundamental misunderstanding of newborn imunity (7, 11). The failed interventions aimed at “correcting” immature immune functions, alongside mounting evidence emerging through next generation sequencing technology, indicate that neonatal immunity exists as a tightly regulated, controlled system which is functionally and purposefully distinct from that of adults, not simply lesser (3, 6–9).

To build the conceptual framework for reconcilation of the clinical observation (increased risk to suffer and die from sepsis in newborns vs. adults) with mechanistic insight regarding host defense in early and adult life, it is necessary to consider the range of host responses to infection that are available. Medzhitov et al. in 2012 outlined three distinct strategies of host defense to infection: disease avoidance, disease tolerance, and disease resistance. In disease avoidance, infection is avoided through behavioral adaptations (e.g., our evolved revulsion to the smell of rotting meat). Disease resistance focuses on the reduction of pathogen burden at the risk of host-inflicted damage (immunopathology). Disease tolerance (DT) strives to minimize immunopathological damage, or fitness cost to the host at the potential cost of unchecked pathogen proliferation (it is important to draw the distinction between “disease tolerance” and “immune tolerance,” which describes regulatory T cell (Treg)-mediated unresponsiveness to potentially immune-activating agents) (12). Animal models have demonstrated newborns to suffer increased mortality when infected with living bacteria (13), viruses (14, 15), or purified inflammatory agonists (16, 17). DT is a well-established concept in biology, but not yet as readily accepted in the human realm (see this entire special edition of *Frontiers*). Specifically regarding DT in early life: newborns are able to withstand a circulating bacterial load 10–100 times greater than adults (<1 CFU per mL blood has been considered to be the clinical “low” threshold in adults, whereas <50 CFU per mL blood has been considered the “low” neonatal threshold; the same trends are observed in animal models) (3, 18). The juxtaposition of increased sensitivity to infection with an enhanced ability to survive greater pathogen loads is the hallmark characteristic of a “disease tolerance” response (12). Yet, as evidenced by the higher burden of infectious disease in newborns, a host defense strategy relying on DT is likely less effective than the adult focus on disease resistance. Despite this clear clinical disadvantge, the newborn host as an organism across evolution appears programmed to more heavily rely on this apparently less-effective DT strategy (3, 19).

We present here a conceptual framework to resolve this conundrum. Based on data demonstrating important links between metabolic pathways and immune functions, it emerges that the pathology of neonatal sepsis is the result of an energy deficit which renders the host incapable of producing critical metabolic mediators to mainain inflammatory homeostasis. While there is an abundance of literature discussing the relationship between organism-level metabolism and cellular immunometabolism in the context of metabolic diseases such as obesity or diabetes (20, 21), the potential impact of organismal metabolic needs has been largely unexplored in the context of infectious disease. Here we hypothesize that the defense strategies differentially employed between newborns and adults (disease tolerance vs. disease resistance) can simply be attributed to differences in systemic energy supply and demand, manifesting at the cellular level as differences in immunometabolic activity.

## Metabolism is fundamentally linked to immunity

Metabolic and immunological functions are intrinsically connected at a level beyond the former simply fueling the latter—metabolic substrates, enzymes, transcription factors, cell receptors, and intermediates have all been shown to have a vast array of immunoregulatory properties. A recent surge in research into this phenomenon (“immunometabolism”) has led to the publication of excellent reviews (22–27) which explore the regulatory role of different metabolic pathways on various leukocytes; with this in mind, we only present a brief overview to introduce the key themes of immunometabolic changes focused on bacterial sepsis.

It has long been known that changes in cellular metabolism occur during sepsis, although until recently these changes were considered to be a result of an oxygen-poor microenvironment due to inflammation-induced hypoperfusion (28). However, there is a large body of evidence indicating that both metabolic shifts and tissue damage in sepsis occur independent of oxygen levels (25, 28–31). TLR activation in certain leukocytes has been shown to activate hypoxia-inducible factor 1α (HIF1α), which upregulates glycolytic pathways and downregulates oxidative phosphorylation—a process known as the Warburg effect (27, 32). This “aerobic glycolysis” is critical to the inflammatory immune response (disease resistance) and represents the primary metabolic activity within immune effector cells (granulocytes, M1 macrophages, cytotoxic, and helper T-cells, NK cells, etc.) (23, 27). While the purpose of switching to aerobic glycolysis in lieu of the more energy efficient process (i.e., ATP-producing) of oxidative phosphorylation is still being debated, it is generally thought that effector cells rely on glycolysis because of one or all of the following reasons: (a) glycolytic intermediates are needed for rapid biosynthesis required for an inflammatory response, (b) glycolysis can be rapidly upregulated and thus can provide a burst of energy faster than oxidative phosphorylation, (c) reactive oxygen species (ROS) are produced during glycolysis which are used in an antimicrobial capacity, (d) glycolysis is better suited to hypoxic/normoxic conditions which may arise during inflammation, and/or (e) increased uptake of glucose minimizes the amount of energy available for invasive bacteria (23, 27, 33). Whatever the reason, it is well established that aerobic glycolysis is a critical component of the disease resistance response (27, 34).

Where aerobic glycolysis is enhanced in effector cells involved in disease resistance pathways, the regulatory and longer lasting cells associated with DT (Tregs, M2 macrophages, memory T cells etc.) increase uptake of exogenous fatty acids and sustain high levels of β-oxidation and oxidative phosphorylation during infection and sepsis (23, 27). While lipids in excess have been shown to induce systemic inflammation (i.e., in obesity), many metabolic intermediates of lipid metabolism exert the opposite effect (35). Circulating lipids which are generated through fatty acid metabolic pathways, namely high-density lipoproteins and very low density lipoproteins, are even capable of directly sequestering LPS and dampening the inflammatory response (36, 37). Lipids belonging to the group of omega 3 fatty acids inhibit the production of inflammatory cytokines and upregulate anti-inflammatory cytokines (38). These lipids also act as precursors to a specialized family of lipids identified as “pro-resolving lipid mediators” (including lipoxins, resolvins, and protectins) which are actively produced to tone down the inflammatory immune response produced at the site of infection (39).

Fatty acid metabolism can therefore be considered to be a fundamental part of the DT response; not only do regulatory / immunosuppressive cells rely on exogenous fatty acids to enact their function, but the metabolites themselves reduce the inflammatory immune response. On the other side of the coin, aerobic glycolysis is a fundamental aspect of the disease resistance response. In addition to the aforementioned increase in glycolytic pathways in immune effector cells, multiple enzymes involved in glycolysis have been shown to either inhibit inflammatory pathways or activate immunosuppressive pathways (27). When a TLR ligand induces high glycolytic flux, these enzymes are rendered incapable of maintaining these disease tolerant functions and the disease resistance response is enhanced (27). To summarize, metabolic shifts in sepsis cannot be separated from inflammatory shifts—the two are fundamentally connected.

## Metabolism in adult sepsis

Metabolic changes during sepsis in adults have been shown to not only be instrumental in diagnosing the disease, but also highly related to survival. A 2013 study (40) of adult patients with community-acquired sepsis examined changes in the plasma metabolome and proteome at time of enrolment and 24 h later. Comparisons were made between survivors (split into three subgroups: uncomplicated sepsis, day 3 severe sepsis, and day 3 septic shock), non-survivors, and a control group of patients exhibiting symptoms but were later determined to have SIRS for non-infectious reasons (SIRS-positive controls). The plasma metabolome revealed four primary findings: (a) the profile of plasma metabolites during sepsis were distinct and reliably distinguishable from SIRS-positive controls, (b) there were marked differences in plasma metabolites between sepsis survivors and non-survivors, (c) there were no differences between the sepsis-survivor subgroups (varying degrees of severity), and (d) there were no major differences between infections caused by *S. pneumoniae, S. aureus, or E. coli* (40). Plasma proteomics mirrored the trend—significant differences between sepsis vs. SIRS-positive control, significant difference between survivors, and non-survivors, minimal (only one) differences within the survivor subgroups, and no significant differences resulting from infections caused by different bacteria. Alterations in fatty acid metabolism largely separated sepsis survivors from non-survivors—the specific pattern of metabolites which were different “suggest a profound defect in β-oxidation in adult sepsis non-survivors that was absent in sepsis survivors” (40).

The authors indicate the above noted differences were not a result of organ dysfunction or hypoxia, but rather due to defects in the process which transports fatty acids from the cytoplasm into the mitochondrial membrane (the carnitine shuttle), which in turn may be attributed to a decrease in peroxisome proliferator-activated receptor-α (PPARα) expression during sepsis. PPARα is the primary transcription factor responsible for controlling a host of genes associated ketone body synthesis (ketogenesis) and transport, a process which in adults is typically associated with prolonged fasting (41). One explanation for the apparent requirement of ketone body production during sepsis is that ketone bodies act as the alternative to glucose for fueling brain metabolic activity, as they are one of the few energetic substrates which are able to cross the blood-brain barrier (42, 43). An animal model examining the impact of exogenous glucose and 2DG (an unmetabolizable analog of glucose which inhibits glycolysis) on sepsis induced by *Listeria monocytogenes*, LPS, influenza virus, and poly(I:C) showed that 2DG's protective effect in bacterial sepsis was mediated through increase in ketogenic activity (PPARα-dependent), which reduced neuronal cell death independent of bacterial load (44). Exogenous glucose alone worsened outcome acting through the same axis—ketone body production was inhibited, and neuronal cell death increased in bacterial sepsis. Curiously, these effects were reversed in the viral sepsis models (poly(I:C) and influenza)−2DG caused 100% mortality and feeding/glucose caused 100% survival, indicating fundamental differences between metabolism during viral and bacterial sepsis.

Another recent study examining longitudinal changes in serum metabolite concentrations during sepsis in adults found non-survivors had elevated (and increasing) levels of TCA cycle metabolites as well as diminished (and declining) numbers of short and long-chain fatty acids (45)—the same trends have previously been described in animal models (46, 47). Though non-survivors in sepsis have diminished fatty acid levels relative to survivors, sepsis itself is generally associated with an increase in plasma lipids (including free fatty acids) when compared to healthy controls (45, 48). Not only do plasma lipids play a critical role in regulating inflammation and providing energy for the brain, but fatty acid metabolism and ketogenesis has also been shown to fuel metabolic activity in many vital organs during active infection (49–51). An impaired capacity for β-oxidation and/or a depleted fatty acid supply will essentially turn off the disease tolerance pathways—death seems almost inevitable through either uncontrolled inflammation or uncontrolled energy expenditure, leaving vital organ functions without fuel. As with any homeostatic environment, poor outcomes are more likely if the balance tips too far to the either extreme.

Furthermore, there is mounting evidence that mortality in adult sepsis is less likely associated with excess inflammation, but rather an immunosuppressive or endotoxin tolerant phenotype (M2-macrophage polarization, anti-inflammatory cytokine production without impaired phagocytic capacity) (52–54). One would expect that as organs begin to fail due to insufficient energy, the body would attempt to increase fatty acid metabolic activity (and inevitably anti-inflammatory activity) at all costs. A prolonged disease resistance response is energetically demanding and eventually it is necessary to revert toward DT by necessity. The heightened death observed in this period may therefore not necessarily be caused by DT, but rather the phenotypic switch to DT as a “last-ditch” effort to adapt to an unsustainable metabolic demand. Perhaps it is time to consider these late-phase inflammatory changes in adult septic patients as a reflection of a different biological mechanism—a slow decrease in the energy available to power vital organ functions.

## Energetic differences in neonates and implications for bacterial sepsis

The implications of the critical role metabolic pathways play in regulating inflammation and providing energy during infection are enormous for newborns, as the energetic demands of growth and development are intense. After adjusting for body weight, healthy newborns require on average three times as much protein (2.2 vs. 0.8 g/kg/day) and more than three times as much total energy (120 vs. 35 kcal/kg/day) as adults (55). Newborns have a lower reservoir of energy, as demonstrated by the percent bodyweight made up of fat (14 vs. 18%) and protein 11 vs. 18%) in neonates and adults (55). Sustaining a controlled immune response requires not only intense glycolytic flux to fuel the cellular proliferation and biosynthesis of disease resistance, but it also requires substantial fatty acid metabolic flux to regulate the inflammation and provide energy to vital tissues. Adults are able to rely on fat and protein stores to provide enough energy to engage in a robust disease resistance response without pulling resources from critical processes, at least until later in infection (see above). This can be observed as up to a 150% increase in resting energy expenditure during bacterial infections in adults (56). Neonates, however, show either no change or even a decrease in resting energy expenditure during sepsis (57–59). An inability to increase energy expenditure relative to the resting state in neonates suggests that the energy to fuel the immune response must be redirected from processes elsewhere in the body. Clearly these processes (likely growth and development) are important enough to warrant maximum energy expenses at a basal state (part of the explanation for relying more heavily on DT than disease resistance). Adults are able to employ the “expensive” disease resistance response without seriously interfering with other vital survival processes; for neonates, any energy spent on immunity has to be “borrowed” from somewhere else. The increased reliance on DT in the newborn allows for less glycolytic flux and thus a lower risk to incur organ failure through an energy deficit during septic episodes.

The first few postnatal days are likely the most energetically demanding period in all of life (60). Immediately after birth, neonates must transition from reliance on maternal glucose to generating it themselves—this manifests as hormonal activation of both glycogenolytic and gluconeogenic pathways in order to rapidly ramp up glucose production to fuel developing organs, especially the brain (60). Further, the newly born infant faces rapid heat loss in the transition from the warm uterine environment to the (relatively) colder external environment. Heat production and oxygen consumption increase two–three-fold within minutes of birth, through both heightened cellular metabolism and non-shivering thermogenesis (metabolism of brown adipose tissue) (61). The high mortality observed on the first day of life in particular may be related to this sudden inability to rely on maternal metabolic and thermoregulatory processes (61, 62). The more energy siphoned toward mounting an immune response, the more sacrifices must be made to fuel the necessary cell proliferation and antimicrobial activities. One would anticipate evolutionary pressures to naturally equilibrate neonatal immunity toward a balance between immunity and development—hence a heightened reliance on DT in neonatal infection.

Metabolomics of the neonatal population have not been studied in nearly as much detail as adults, though what is available indicates that metabolism is a critical component of neonatal sepsis as well. A transcriptomic comparison of newborns with bacterial sepsis against healthy controls was used to construct a classifier that accurately identified septic neonates; inclusion of only genes which were associated with standard immune functions (inflammation, etc.) resulted in a classifier with 100% sensitivity but less than 30% specificity, but the inclusion of metabolic genes brought the specificity up to 100% (6). Specifically, they showed that bacterial sepsis in neonates is associated with increased expression of genes related to glycolysis (glucose transporter GLUT3, glycolysis activator PFKFB3, and initiating hexokinase HK3), fatty acid metabolism and metabolic homeostasis (principally via regulatory STAT3 and receptor FFAR2). In the validation test set of the classifier, the three instances of viral sepsis clustered with the 6 controls; the viral patients did not show the distinct metabolic profile which was so visible in newbons with bacterial sepsis, further indicating that uncontrolled viral proliferation has a profoundly different impact on the body than uncontrolled bacterial proliferation (6).

As mentioned above, poor outcomes in adult sepsis correlate with an inhibited ability to produce ketone bodies via PPARα. While ketone body metabolism in the adult brain is typically reserved for a starvation response (which perhaps should be updated to “energetically demanding periods” such as sepsis), there is evidence from animal models that neonates rely on ketone bodies as an energy source in the brain independent of starvation (43). Specifically, newborn rats rely on ketone bodies for up to 40% of the energy production in the brain (42) and newborn cynomolgus monkeys exhibited increased expression of blood-brain barrier ketone body transporter protein MCT1, with levels decreasing as a function of age (plateauing in adulthood) (63). The process of birth necessitates a series of metabolic adaptations from receiving nutrients via the placenta (high-carbohydrate, low-fat) to receiving nutrients via breastmilk (low-carbohydrate, high-fat) (64, 65). One manifestation of these adaptations is the activation of PPARα immediately prior to birth, presumably in anticipation of the new, fat-rich diet (64). Upon the switch to breastmilk, neonates rely on ketone bodies to fuel brain activity which allows glucose (broken down from lactose) to enter the pentose phosphate pathway, producing the nucleic acids and lipids necessary for cerebral growth (43).

Similarly, a metabolomic analysis of urine from septic newborns indicated a substantial increase in acetone ketone bodies (and other byproducts of fatty acid oxidation) relative to healthy controls (66). If more ketone bodies are found outside of the brain but there is little compensatory increase in ketone body production [as indicated by animal models (47), and neonates being “maxed-out” in their energy expenditure at baseline], then one can assume that ketone bodies which are needed in the brain are being deployed elsewhere in the body, and hence the brain is running at an energetic deficit. This is just one example of the type of vital process which may be interrupted by mounting an immune response during bacterial sepsis.

## Metabolism in viral sepsis

As eluded to above, the metabolic signatures specific to bacterial sepsis appear to be absent in viral sepsis; gene signatures from human newborns that predict bacterial sepsis classify viral patients as controls (6) and in an experimental model, inhibiting glucose metabolism led to 100% mortality viral infection and poly(I:C) challenge (44). Hence, metabolic regulation during viral infection is likely distinct from that of bacterial infection. This is not of small consequence, as a substantial proportion of neonatal sepsis may be due to viruses: In a published unit from a Bangladeshi cohort, 36% of suspected neonatal sepsis was viral (67), though it is difficult to estimate the generalizability. Viruses rely on cellular fatty acid synthesis in order to replicate, which is reflected by viral manipulation of cellular metabolism. Human cytolomegavirus has been shown to sequester glucose from the the TCA cycle and redirect it toward virus-induced fatty acid biosynthesis, demonstrating just one example of the potential immunometabolic ramifications of viral infection (68). Inhibiting fatty acid biosynthesis has been shown to slow the growth of viruses; one study showed that treating cells with in inhibitor of fatty acid synthase reduced the proliferation of rotavirus (69). Similarly, AMP-activated kinase is able to decrease Rift Valley virus infection by inhibiting cellular fatty acid synthesis necessary for viral replication (70). Meanwhile, short-chain fatty acids derived from dietary fiber are protective against influenza challenge due to their effect on both innate and adaptive immune cells (71). Thus, strategically manipulating the glucose/fatty acid metabolism has potential in improving outcomes in newborn viral sepsis and need to be studied further in this context. Already, differences in feeding patterns during viral and bacterial infection may hint at the unique nutritional requirements for both types of infections. Poor feeding itself is a hallmark of newborn infection (72). However, it seems to be more prominent during bacterial, compared to viral sepsis: in a cohort of febrile newborns with enterovirus, ~16% had poor feeding (73) while in another cohort of Ugandan infants with or without culture-positive sepsis, 42% of newborns with culture positive sepsis (i.e., bacterial) were admitted for care with poor feeding as a primary sign, compared to only 17% of infants admitted with a diagnosis of culture negative sepsis (i.e., viral) (74). Clearly more research is warranted into the immunometabolic differences between bacterial and viral sepsis.

## Nutritional therapy and the microbiome

If mortality in bacterial sepsis can be attributed to an energetic deficiency, then one must be able to explain how nutritional supplementation (a standard practice in any ICU or NICU) does not represent the most effective sepsis treatment. As with everything else in sepsis, the efficacy of feeding as an intervention is limited by its ability to maintain homeostasis. The previously described study by Wang et al. where inhibition of glycolysis led to 100% survival (and feeding led to 100% mortality) in adult, LPS-challenged mice challenged provides an excellent example of nutritional supplementation creating a homeostatic imbalance and leading to negative outcome. Both exogenous glucose and food gavage inhibited ketogenesis which led to an energetic imbalance (glucose being siphoned into the immune response with no ketone bodies to replace it) and an inflammatory imbalance (diminished anti-inflammatory lipid mediators). One also must consider the dangers of overfeeding—overfeeding has been shown to worsen sepsis outcomes in both animal models and human observational studies due to hyperglycemia, elevated inflammatory markers, dysregulated immune responses, and presumably enhanced nutrients for pathogen growth (75–78). Further, this hypothesis poses that the metabolic risk comes from a shift in the proportion of energy expended toward disease resistance pathways over maintaining organ function—the danger is not only tied to the overall capacity, but the utilization of energy present. If a system has reached the point where it is spending 100% of its resources on fighting infection, no amount of exogenous nutrients will make a difference (unless accompanied by a simultaneous change in resource allocation).

Early enteral nutrition (EN) in adult patients with prolonged sepsis has been shown to improve patient outcomes, reduce oxidative stress, improve gut epithelial integrity, and downregulate systemic immune responses (79); correspondingly, negative energy balance has been shown to be associated with worse clinical outcomes (80). EN not only addresses the caloric deficit which is inevitable in prolonged sepsis but seems to modulate immune functions through interfacing with the gut-associated lymphoid tissue and upregulating Th2 cell proliferation. Parenteral nutrition (PN) has been shown to be less effective than EN—a 2013 study by Elke et al. found that rate of death was significantly lower in adult ICU patients with sepsis which received EN rather than PN or EN + PN combined (26.7 vs. 41.3%); in addition to this mortality reduction, duration of mechanical ventilation and rate of secondary infection were also decreased in the EN-alone group (81). Increased mortality from PN (relative to EN) is thought to be due to a heightening of the inflammatory response associated with hyperglycemia, an effect which exacerbated in PN due to bypassing metabolic regulatory axes associated with the GI tract (79). This has interesting implications for neonates, where the nascent microbiome represents another axis which is distinct from adults (82). The diet of neonates (lactate-heavy breastmilk) results in a colonization pattern of commensal bacteria which fascilitate nutrient absorption and produce a wide array of immunoregulatory metabolites (82, 83). The limited biodiversity present in the neonatal microbiome could result in an impaired ability to incorporate nutrients without altering inflammatory homeostasis—any inability to control the potential energy flux of disease resistance represents another potential explanation for the neonatal reliance on DT.

## Recontextualizing newborn immunity

The implications of this hypothesis are broad and may explain other aspects neonatal immunity. Newborns have been described as exhibiting an immunosuppressive phenotype, which has often been considered to be a vestige of time spent *in utero* where active fetal immunity could result in miscarriage (8, 84). This alternative hypothesis to DT fails to explain the persistance of many of these immunosuppressive actors well after the first few days of life. For example, neonatal myeloid-derived suppressor cells and anti-inflammatory CD5^+^ B cells remain significantly higher than adult levels for more than 6 months and 4 months after birth, respectively (8, 85). Given the high burden of infectious disease in early life, one would anticipate evolutionary pressure to drive the time spend in this “anti-inflammatory phase” to as little as possible. If, however, neonatal immunity is limited by an availability of energy, then it would be critical to maintain some immunosuppressive cells to limit the magnitude of an inflammatory response until the body is able to better sustain it. While the “fetal suppression” hypothesis may in part explain the susceptibility of term infants to bacterial sepsis (86), it seems unlikely that a biological liability of this magnitude (suppressed immune system) would exist and persist if it did not convey some sort of survival advantage (DT).

The extreme susceptibility to infection observed in preterm newborns may be in part due to the extreme energy demands associated with survival and rapid development, but it is more difficult to discount alternative explanations such as immaturity and immune suppression to tolerate maternal antigens. As outlined in a recent review by Collins et al. susceptibility of preterm newborns to infection can be attributed to “comprimised [innate] barriers, inflammatory response elements, and cells”—more research is warranted to elucidate the role metabolic demands play in preterm immunity (87).

## Summary

**Figure 1:**
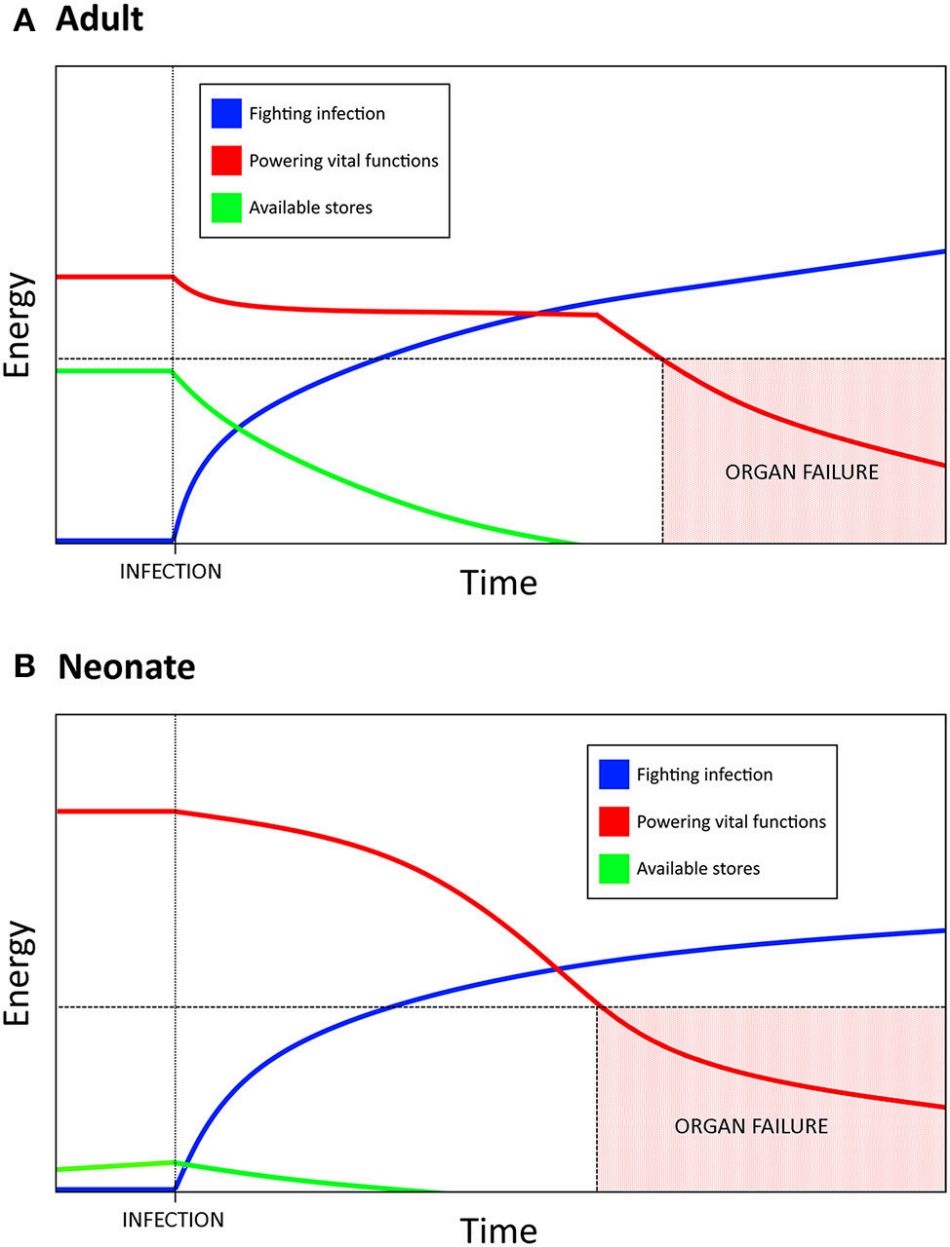
Resource allocation in bacterial sepsis explains the need for disease tolerance as a defense strategy in neonates. Bacterial infection requires a massive, sustained energy input in order to mount an inflammatory, disease resistance response. **(A)** Adults have substantial energy stores and a resting metabolic rate that is not near its max capacity, which allows for a burst of energy to be rapidly allocated toward fighting the infection. If the infection results in a prolonged state of sepsis, eventually the amount of energy required to maintain the inflammatory response necessitates a siphoning from other vital functions. Too much energy siphoned can result in organ failure and lead to mortality. **(B)** Neonates have much higher basal metabolic needs, lower energy stores than adults, and are unable to increase resting energy expenditure during infection. Allocating resources toward fighting infection therefore comes at a greater cost to neonates, which has resulted in an increased reliance on disease tolerance as a defense strategy. Mortality due to sepsis may be a result of the energetic demands of a sustained response, rather than a direct result uncontrolled inflammation.

Mortality in sepsis has been attributed to a dysregulated inflammatory response, with the current paradigm indicating that death may be the result of straying too far toward either extreme (88). Here we hypothesize inflammation is only the top layer of this process and that it is an underlying mechanism, namely metabolism, which is the driving force behind mortality in sepsis. Through this paradigm, the neonatal reliance on DT as a host defense strategy is much easier to understand—the newborn response to sepsis can be characterized through the distinct metabolic needs unique to early life. Lack of functional fat stores, resting metabolic rate operating near or at 100% of its potential capacity, and heavy activation of PPARα due to the high fat content of breast milk combine to constrict the magnitude of the neonatal potential for a disease resistance response (i.e., inflammation). As glycolysis is fundamentally tied to effector/antimicrobial/disease resistance cell functions, it becomes necessary to shift as much glycolytic activity toward fighting infection as possible. The body then transitions into the equivalent of a “starvation state” where ketogenesis is used to support vital organ functions, especially in the brain as there is no other alternative energy source which is able to permeate the blood brain barrier (51). Birth itself overlaps with this “starvation state” in that the process of adapting from *in utero* to *ex utero* life is energetically intense. This creates a situation where neonates are unable to produce the burst of energy associated with aerobic glycolysis which is characteristic of adult immunity—powering a full disease resistance strategy would mean pulling resources from elsewhere in the newborn body. The more intense and prolonged this response is, the more likely it is that a vital organ will fail as there simply is not enough energy devoted to maintaining its functions (Figure 1).

Homeostasis in sepsis thus cannot be considered to only be a balance between inflammatory (disease resistance) and immunosuppressive (disease tolerance) processes, but rather a balance of inflammation and aerobic glycolysis (disease resistance) with immunosuppression and fatty acid metabolism (disease tolerance). The less excess energy which exists in the system as a whole, the more an individual must rely on the DT approach. This applies not only to neonates, but also to adults facing prolonged infection. Nutritional supplementation is critical to survival, but it must be provided within the bounds of this homeostatic balance. Excess nutrients or parenteral nutrition would impair ketogenesis which would (a) remove anti-inflammatory lipid substrates/metabolites resulting in excess inflammation, (b) diminish the amount of energy devoted to fuel vital functions (increased inflammation resulting in increased glycolysis which is being used to fight infection rather than support organ functions), and (c) provide resources for the invasive pathogen and not only the host. Impairing glycolysis, on the other hand, runs the risk of impairing the entire disease resistance branch and exposing the host to the danger of uncontrolled pathogen proliferation. It is critial that future interventions which are theoretically focused on managing inflammation also consider the potential impact on energy homeostasis, and interventions aimed at nutritional supplementation (both prenatal and perinatal) must not disrupt inflammatory homeostasis. More research in novel therapeutics which act on both fronts is clearly warranted.

## Author contributions

All authors contributed to the conceptual idea and the editing process. DH made the figure and wrote most of the manuscript with writing contributions from NA and FF, as well as guidance from NA and TK. FF and WB provided research assistance.

### Conflict of interest statement

The authors declare that the research was conducted in the absence of any commercial or financial relationships that could be construed as a potential conflict of interest.
